# Comparative durability of pacemaker leads in transvenous lead extraction: An evaluation through bench testing

**DOI:** 10.1016/j.hroo.2025.01.015

**Published:** 2025-01-30

**Authors:** Junji Morita, Ayako Okada, Fred Kusumoto, Kentaro Nakamura

**Affiliations:** 1 Department of Cardiovascular Medicine, Sapporo Cardiovascular Clinic, Sapporo, Hokkaido, Japan; 2 Department of Cardiovascular Medicine, Shinshu University School of Medicine, Nagano, Japan; 3 Department of Cardiovascular Medicine, Heart Rhythm Division, Mayo Clinic, Jacksonville, Florida; 4 Department of Cardiology, Urasoe General Hospital, Urasoe, Okinawa, Japan

**Keywords:** Lead durability, Pacemaker leads, Snare technique, Tensile testing, Terminal retention, Transvenous lead extraction

## Abstract

**Background:**

Transvenous lead extraction (TLE) is a less invasive alternative to surgical removal, but leads may break during TLE.

**Objective:**

The purpose of this study was to compare the tensile strength and behavior under stress of leads from different manufacturers during TLE using different extraction techniques.

**Methods:**

Different lead types (Tendril STS, INGEVITY, INGEVITY+, Solia S, and CapSure Fix) were subjected to tensile testing using a testing machine. The leads were tested in up to 3 different configurations: terminal removed, terminal retained, and removal using a snare. Lead durability was assessed by increasing force and measuring elongation and the force required for complete disruption.

**Results:**

The tensile strength of leads varied greatly based on the extraction technique and lead type. The INGEVITY leads showed improved durability when secured with a snare, despite weak performance when the terminal was removed or retained. The INGEVITY+ lead exhibited enhanced durability, particularly when removed using a snare. The Tendril STS lead exhibited the least durability when the terminal was removed but had comparable durability to the Solia S and CapSure Fix leads when the terminal was retained. The Solia S and CapSure Fix leads had consistent higher durability, irrespective of whether or not the terminal was removed.

**Conclusion:**

This study found wide variability of lead behavior among manufacturers and with different extraction techniques. Terminal retention and snare usage enhanced lead durability, suggesting that these techniques should be considered depending on lead type and for leads with longer implantation periods or if higher extraction forces are anticipated.

**Key Findings**

▪ Terminal retention increased lead durability, whereas snare grasping further enhanced durability.
▪ The lead terminal ideally should be retained during transvenous lead extraction (TLE) of a Tendril STS lead, and if a high traction force is anticipated because of clinical characteristics, a snare should be considered as an initial approach or adopted early during lead extraction.
▪ A snare should be used in most TLE cases involving an INGEVITY lead. For the INGEVITY+ lead, the terminal should be retained and a snare considered if higher traction forces are anticipated.

## Introduction

Cardiac implantable electronic devices are widely used for treating selected patients with heart failure and rhythm disorders, with 1.2–1.4 million patients receiving implants annually.^1^, ^2^, ^3^ Consequently, the number of lead explants or extractions necessitated by infections caused by cardiac implantable electronic devices, lead malfunctions, and recalls has increased, amounting to approximately 30,000 cases per year.^3^^,^^4^ Compared to surgical lead removal, transvenous lead extraction (TLE) is less invasive and is associated with a lower risk of complications.^5^, ^6^, ^7^, ^8^ When more than 40 mm of lead remains after an extraction, the procedure is considered to be unsuccessful.^3^ In addition, lead breakage during the extraction procedure can complicate the procedure.^9^, ^10^, ^11^

Traditional TLE techniques using a powered sheath involve the removal of the terminal portion of the lead. However, retaining the terminal end of the INGEVITY lead (Boston Scientific, St. Paul, MN) and using a snare has been reported to increase its tensile strength.^12^ Because the structure of the leads varies across manufacturers and individual leads, their durability with traction is likely to differ. However, comparisons of various leads have not been reported to date.

In this bench test study, we compared the tensile strength and behavior under stress of leads from different manufacturers, using various approaches, including terminal removal, terminal retention, and terminal removed with use of a snare.

## Methods

**Figure 1:**
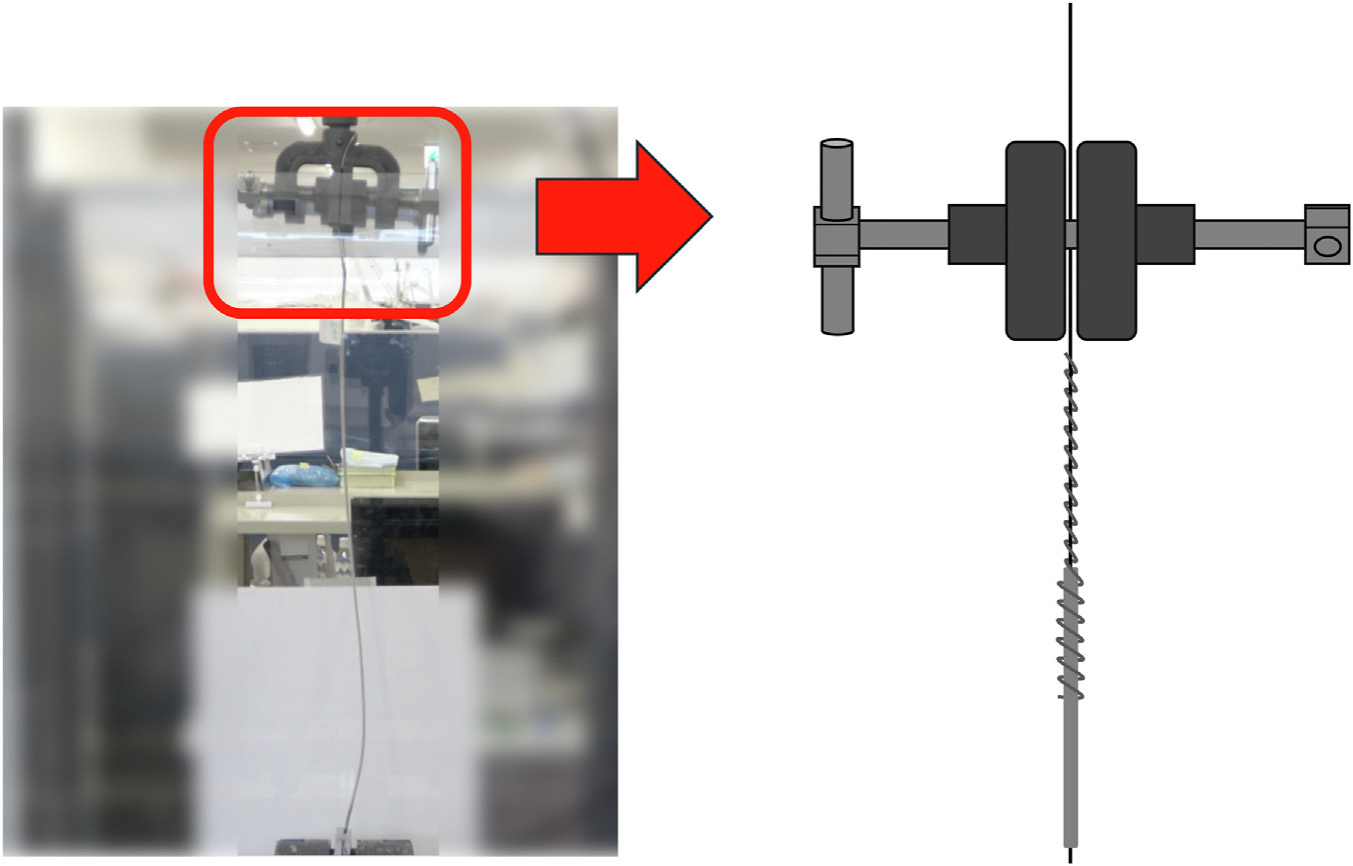
The terminal pin side of the lead was cut, and this group was named the "terminal-removed group." This approach aligns with traditional transvenous lead extraction techniques, in which the terminal pin side of the lead is cut. The cut end and the lead were wrapped with a One-tie.

**Figure 2:**
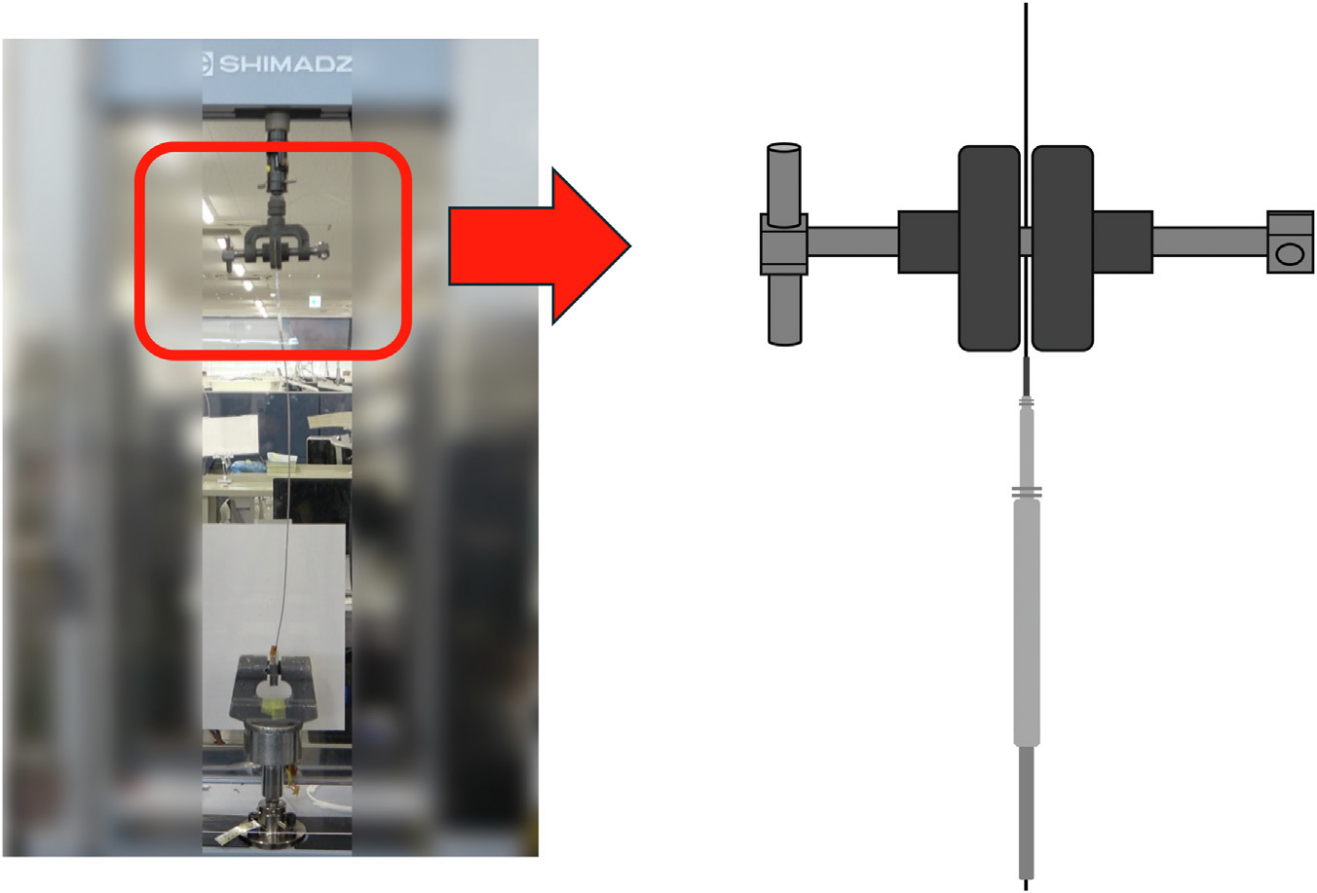
The terminal pin side of the lead was not cut, and a Liberator was inserted with the One-Tie wrapped around it. This is the terminal-retained group.

**Figure 3:**
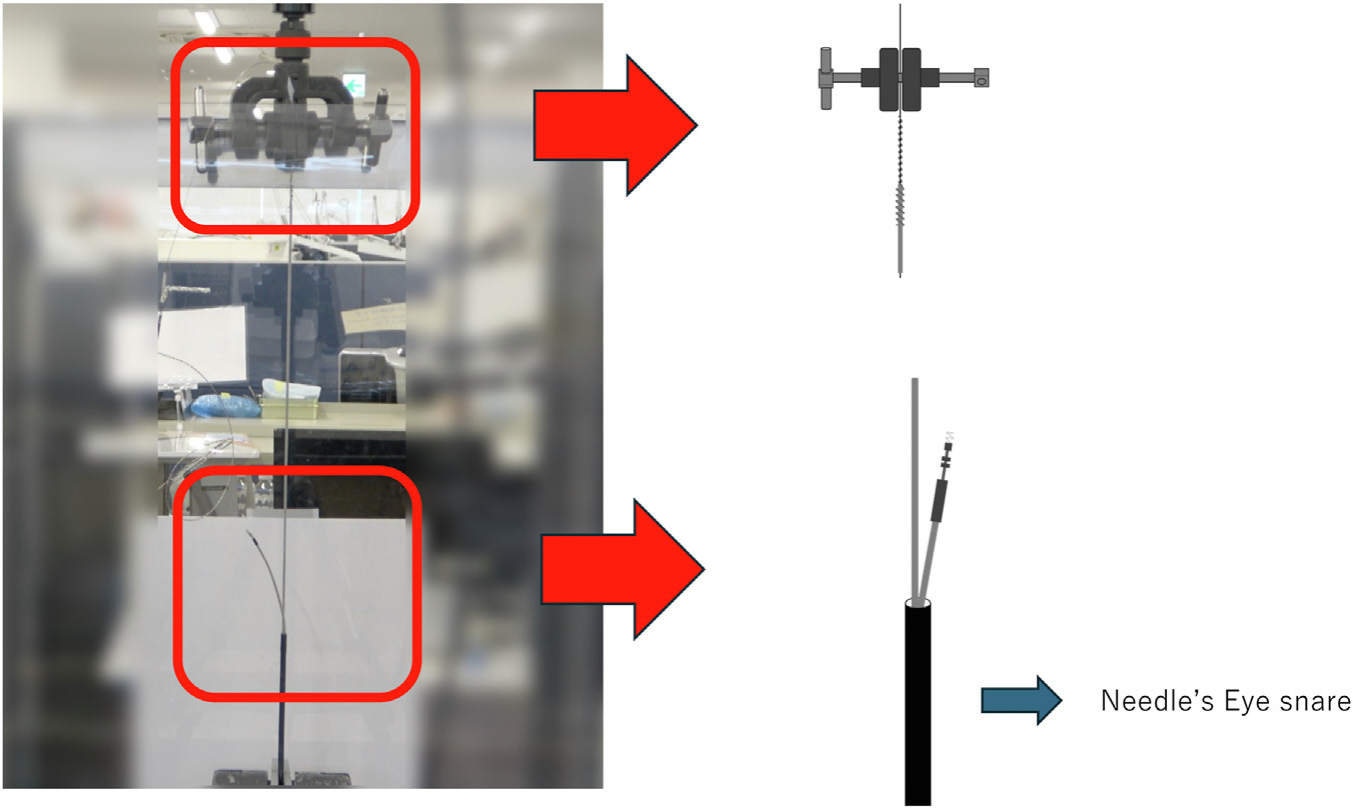
In the snare group, the terminal pin side of the lead was cut, a Liberator inserted, and the One-Tie wrapped around the lead. The lead was gripped 6 cm from the tip with a Needle’s Eye Snare (Cook Medical). The snare shaft was clamped and secured to the test machine.

We used Tendril STS (active fixation; Abbott, Abbott Park, IL), INGEVITY (active fixation; Boston Scientific), INGEVITY+ (active fixation; Boston Scientific), Solia S (active fixation; Biotronik, Berlin, Germany), and CapSure Fix (active fixation; Medtronic, Minneapolis, MN). The groups were defined as follows: the group in which the terminal pin side of the lead was cut was named the "terminal-removed group"; the group in which the terminal pin side of the lead was not cut was named the "terminal-retained group"; and the group in which a snare was used was named the "snare group." Tendril STS, INGEVITY, and INGEVITY+ leads were tested in 3 categories: terminal removed, terminal retained, and terminal removed using a snare. Solia S and CapSure Fix leads were tested in only 2 categories: terminal removed and terminal retained. The leads were subjected to tensile testing to measure their durability using a Shimadzu AGS-X 5 kN load cell tensile testing machine (Shimadzu Corp., Kyoto, Japan). The leads were prepared to simulate clinical TLE procedures closely (Figure 1, Figure 2, Figure 3).

For the terminal-removed group, the terminal pin side of the lead was cut, and a locking stylet (Liberator, Cook Medical, Bloomington, IN) was inserted and the insulation secured with a One-Tie (Cook Medical) wrapped around the lead (Figure 1 and Supplemental Video 1). For the terminal-retained group, the terminal pin side of the lead was not cut, and a locking stylet was inserted and a One-Tie wrapped around it (Figure 2 and Supplemental Video 2). In both groups, the lead tip was secured to the lower part of the machine and the locking stylet to the upper part of the machine. For the snare group, the terminal pin side of the lead was cut, a Liberator was inserted to the tip of the leads, and the One-Tie was wrapped around the lead.

**Figure 4:**
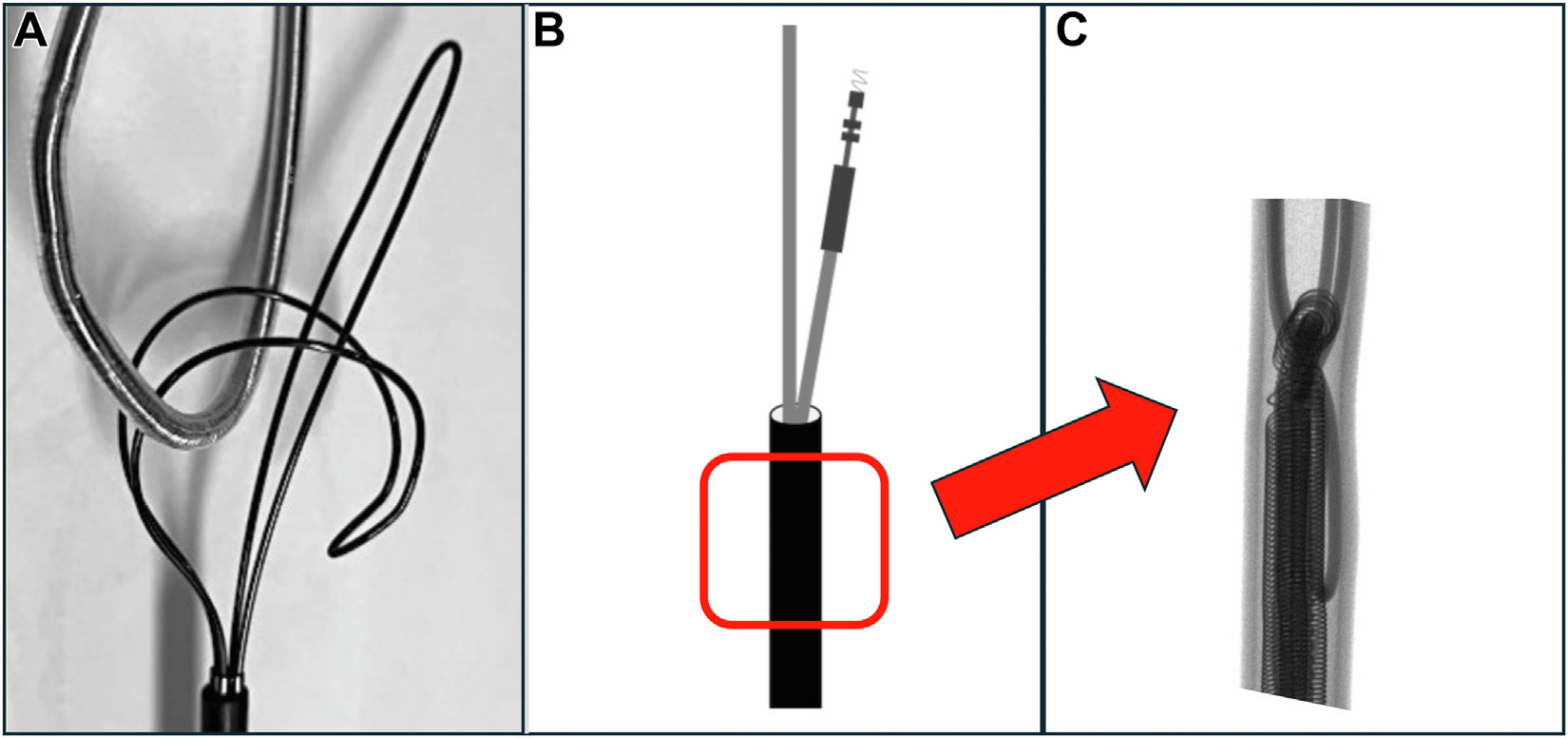
The lead was gripped 6 cm (2.36 in) from the tip using a needle’s eye snare **(A),** and this combined structure was buckled into the sheath **(B). C:** The locking stylet and the lead are simultaneously grasped by the snare.

The lead was gripped 6 cm (2.36 in) from the tip using a Needle’s Eye Snare (Cook Medical) (Figure 3 and Supplemental Video 3). The locking stylet and the lead were simultaneously grasped by the snare and buckled into the sheath (Figure 4). The snare shaft was secured to the lower part of the test machine. For all groups, the upper part of the testing machine was continuously pulled upward at a constant speed to evaluate the durability of the lead based on the force and distance applied to the machine. Due to size constraints of the machine, the terminal-removed group was pulled to a maximum of 300 mm (11.81 in), whereas the terminal-retained and snare groups were pulled to a maximum of 100 mm (3.94 in). X-ray imaging of the lead was performed before and after testing to evaluate the internal structure.

## Results

### Terminal-removed group

**Figure 5:**
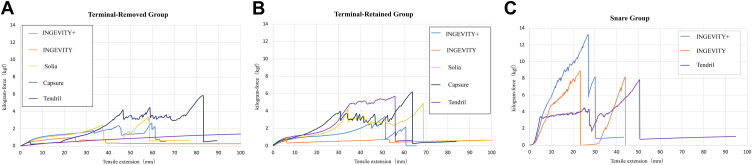
**A:** In the terminal-removed group, the Tendril STS lead required up to 1.4 kgf (3.09 lbf) to pull 100 mm (3.94 in) without snapping. The INGEVITY+ and Solia S leads snapped at around 60 mm (2.36 in) with forces of 2.7 kgf (5.95 lbf) and 3.4 kgf (7.5 lbf), respectively. The CapSure Fix lead snapped at 83 mm (3.27 in) with a force of 5.9 kgf (13.0 lbf). **B:** In the terminal-retained group, the INGEVITY lead could be pulled 100 mm (3.94 in) with <1 kgf (2.2 lbf), whereas other leads showed higher forces. The Tendril STS lead snapped at 35 mm (1.38 in) with 4.9 kgf (10.8 lbf), and the INGEVITY+ lead snapped at 61 mm (2.4 in) with 2.2 kgf (4.85 lbf). The Solia S lead snapped at 69 mm (2.72 in) with 4.9 kgf (10.8 lbf), and the CapSure Fix lead snapped at 64 mm (2.52 in) with 6.2 kgf (13.67 lbf). **C:** In the snare group, the Tendril STS lead snapped at 50.5 mm (1.99 in) with 7.8 kgf (17.2 lbf), the INGEVITY lead at 44 mm (1.73 in) with 8.2 kgf (18.1 lbf), and the INGEVITY+ lead at 30 mm (1.18 in) with 13.2 kgf (29.1 lbf).

In the terminal-removed group, the Tendril STS and INGEVITY leads exhibited similar curves (Figure 5A). Both leads elongated over 50 mm (1.97 in) with a force <1 kgf (2.2 lbf). For the INGEVITY lead, a force of 0.7 kgf (1.54 lbf) was required to pull it 10 mm (0.39 in), 1 kgf (2.2 lbf) to pull it 25 mm (0.98 in), and could be pulled to 280 mm (11.02 in) by 1.2 kgf (2.65 lbf) before it could be completely broken by the cathode coil breaking. The Tendril STS lead required approximately 0.3 kgf (0.66 lbf) to pull it 10 mm (0.39 in) and 0.6 kgf (1.32 lbf) to pull it 30 mm (1.18 in). A force of 1.4 kgf (3.09 lbf) could pull it a distance of 100 mm (3.94 in). This lead was pulled to 300 mm (11.81 in) without a complete break with a final force of 2.7 kgf (5.95 lbf).

The INGEVITY+ and Solia S leads showed similar curves. The INGEVITY+ lead required 2.3 kgf (5.07 lbf) to pull it 45 mm (1.77 in), at which point a partial break occurred between the cathode coil and the anode electrode, accompanied by a popping sound, and the force decreased. When pulled again, the force increased to 2.7 kgf (5.95 lbf) at 60 mm (2.36 in), after which a complete break occurred with cathode coil breaking. The Solia S lead required 2.4 kgf (5.29 lbf) to pull it 37 mm (1.46 in), at which point a partial break occurred between the cathode coil and the anode electrode, accompanied by a popping sound and the force decreased. Subsequently, the force continued to increase, reaching 3.4 kgf (7.5 lbf) at 58 mm (2.28 in), at which point a complete break occurred with the cathode coil breaking.

The CapSure Fix lead exhibited a different type of curve. To pull it 40 mm (1.57 in), 2 kgf (4.41 lbf) was required, similar to the Solia S and INGEVITY+ leads. However, it did not produce a popping sound or a sudden decrease in force. Instead, the force increased to 4.2 kgf (9.26 lbf) by 46 mm (1.81 in), at which point a partial break occurred between the cathode coil and the anode electrode, accompanied by a popping sound. The force then decreased but began to increase again from 75 mm (2.95 in), reaching 5.9 kgf (13.0 lbf) at 83 mm (3.27 in), at which point a complete break occurred.

### Terminal-retained group

For the INGEVITY lead, a force of 0.7 kgf (1.54 lbf) was required to pull it 53 mm (2.09 in), and a partial break occurred between the cathode coil and the anode electrode, accompanied by a popping sound, after which the force decreased. Subsequently, the lead was pulled to 100 mm (3.94 in) using a force <0.7 kgf (1.54 lbf). In contrast to the terminal-removed context, the Tendril STS lead was pulled for <10 mm (0.39 in) by a force of 1 kgf (2.2 lbf). To reach 35 mm (1.38 in), a force of 4.9 kgf (10.8 lbf) was required, resulting in a partial break accompanied by a popping sound. However, the force continued to increase gradually thereafter. At 5.7 kgf (12.57 lbf), a second popping sound was heard, followed by complete break of the lead. The INGEVITY+ lead exhibited a gradual increase in force, reaching 3.4 kgf (7.5 lbf) at 51 mm (2.01 in). At this point, a partial break occurred between the cathode coil and the anode electrode, and the force decreased. To pull it further to 61 mm (2.4 in), a force of 2.2 kgf (4.85 lbf) was applied, at which point the lead completely broke.

The Solia S and CapSure Fix leads displayed similar graph curves. The Solia S lead required a force of 4.2 kgf (9.26 lbf) to reach 38 mm (1.50 in), after which a partial break occurred between the cathode coil and the anode electrode, followed by a decrease in measured force. Continued pulling resulted in an undulating curve with the force remaining stable up to 55 mm (2.17 in). At 55 mm (2.17 in), the force began to rise again, reaching 4.9 kgf (10.8 lbf) at 69 mm (2.72 in), before the lead completely broke. The CapSure Fix lead required a force of 4.1 kgf (9.04 lbf) to reach 31 mm (1.22 in). The force decreased slightly thereafter but increased again, reaching 6.2 kgf (13.67 lbf) at 64 mm (2.52 in), before the lead completely broke.

### Snare group

**Figure 6:**
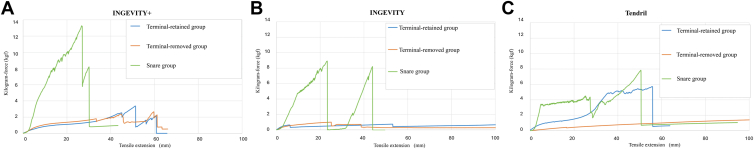
Comparison of the INGEVITY+ **(A),** INGEVITY **(B),** and Tendril STS **(C)** leads on a per-lead basis. Use of a snare with the INGEVITY+ and INGEVITY leads shows significantly greater force compared to terminal-removed and terminal-retained. For the Tendril STS lead, the force with terminal-retained and snare is significantly greater compared to terminal-removed.

The Tendril STS, INGEVITY, and INGEVITY+ leads were assessed for TLE with a snare, and all demonstrated increased lead tolerance compared to other extraction strategies (Figure 6). The Tendril STS lead required a force of 7.8 kgf (17.2 lbf) to elongate to 50.5 mm (1.99 in), at which point the lead completely broke. The INGEVITY lead required a force of 8.9 kgf (19.6 lbf) to reach 23 mm (0.91 in), after which the lead completely broke. Subsequently, the force dropped to 0.1 kgf (0.22 lbf) but began to increase again from 31 mm (1.22 in). At 44 mm (1.73 in), the force rose to 8.2 kgf (18.1 lbf), after which the lead completely broke. The INGEVITY+ lead required a force of 13.2 kgf (29.1 lbf) at 27 mm (1.06 in), accompanied by a popping sound and a sudden decrease to 5.9 kgf (13.0 lbf). Further pulling to 30 mm (1.18 in) resulted in a complete break.

### Radiographic data

**Figure 7:**
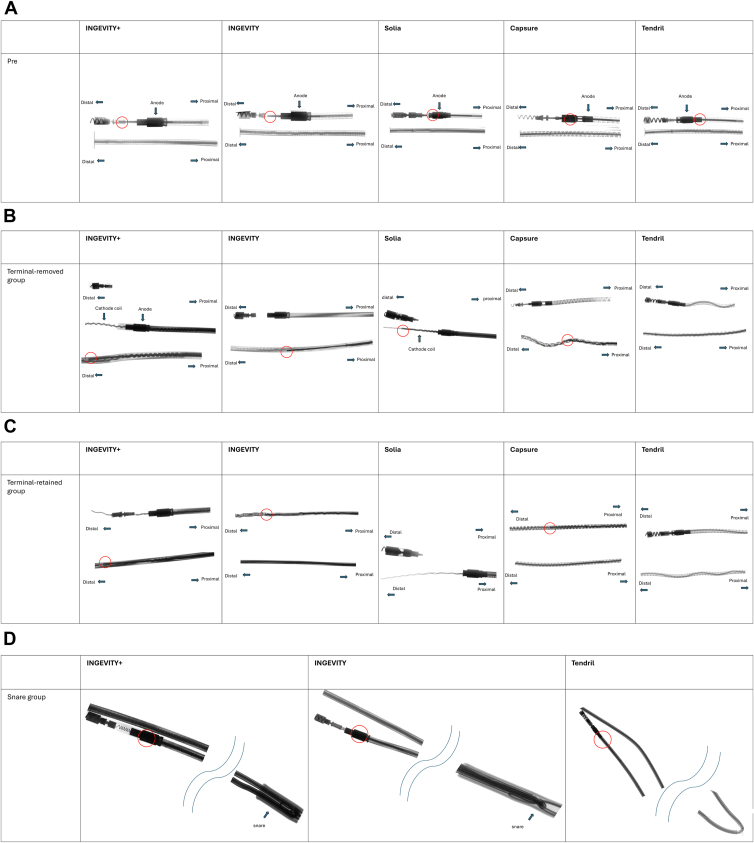
**A:** X-ray image after inserting and locking the locking stylet, before traction. The *red circle* indicates the tip of the locking stylet. For the Tendril STS, the tip of the locking stylet was positioned proximal to the anode. **B:** X-ray image after the traction test in the terminal-removed group. The *red circle* indicates the tip of the locking stylet. In INGEVITY+, the cathode coil extended distally beyond the locking stylet. INGEVITY showed more prominent extension of the cathode coil than INGEVITY+. The cathode coil extended even in the part where the locking stylet is inserted. In Solia S, the locking stylet can be seen within the cathode coil distal to the anode, indicating relatively less sliding of the locking stylet. In CapSure Fix and Tendril STS, the upper shows the distal area without the locking stylet, whereas the lower shows the proximal area with the locking stylet inserted. CapSure Fix did not show the cathode coil in the distal part. In the area without the locking stylet, the cathode coil was extended. In Tendril STS, the cathode coil remained in the distal part, extending uniformly compared with other leads. **C:** X-ray image after the traction test in the terminal-retained group. The *red circle* indicates the tip of the locking stylet. Each lead exhibited the same extension as observed when the terminal was removed. Unlike when the terminal was removed, Solia S did not show the locking stylet distal to the anode. **D:** X-ray image after the traction test in the snare group. For Tendril STS, because the snare came off, X-ray images of the bent area were taken similar to that for INGEVITY. Each lead maintained structural balance compared with the terminal-removed and terminal-retained groups.

X-ray images were obtained before and after the pulling experiments to assess the degree of rupture in the internal structure of the leads (Figure 7). Figure 7A shows the pretesting X-ray images and shows that the tip of the locking stylet extends distal to the anode in all leads except the Tendril STS. Figure 7B shows X-ray images after the traction test in the terminal-removed group. In INGEVITY+, the cathode coil extended distally beyond the locking stylet. It is evident that the cathode coil extends, particularly due to its longer length distal to the anode. INGEVITY showed more prominent extension of the cathode coil than INGEVITY+. The cathode coil extended even in the part where the locking stylet is inserted. In Solia S, the locking stylet can be seen within the cathode coil distal to the anode, indicating relatively less sliding of the locking stylet. In CapSure Fix and Tendril STS, the upper shows the distal area without the locking stylet, while the lower shows the proximal area with the locking stylet inserted. CapSure Fix did not show the cathode coil in the distal part. In the area without the locking stylet, the cathode coil was stretched. In Tendril STS, the locking stylet was not observed, suggesting that extensive slippage of the locking stylet in addition to stretching of the coils were the explanation for its behavior. Figure 7C shows X-ray images after the traction test in the terminal-retained group. Each lead exhibited the same total extension as observed when the terminal was removed and similar radiographic findings. Figure 7D shows X-ray images after the traction test in the snare group. For INGEVITY+ and INGEVITY, the snare did not come off, so X-ray images were taken with the snare grasping the leads. For Tendril STS, because the snare came off, X-ray images were taken of the bent area similar to INGEVITY. Each lead maintained structural balance compared with terminal-removed and terminal-retained groups. Additionally, there was no observed slippage of the locking stylet.

The internal coils were found to have unraveled and extended. The terminal-removed group exhibited the most coil unraveling, followed by the terminal-retained group, with the snare group showing the least coil unraveling. In the snare group, the snare-gripping section was firmly gripped.

## Discussion

We compared TLE of leads from different manufacturers by using terminal removal, terminal retaining, and terminal removal with a snare approaches, and compared the force required to pull the lead over a certain distance before it completely broke, using a tensile testing machine. This bench test study yielded several significant results.

### Concept of the TLE technique

During lead extraction, if the lead adheres to a blood vessel, a powered sheath is used to separate the adhesions. As the sheath advances, force is required for such separation. If the traction on the lead is insufficient, it can bend, fail to pass through, and could contact the vessel wall, risking vascular damage. Thus, proper traction is crucial. The balance between the force applied to advance the sheath and the traction applied to the lead determines whether the lead will bend.

With strong adhesions, greater traction on the lead is needed, but too much force can cause lead breakage, which would hamper maintenance of coaxial alignment and complicate extractions. Lead breakage can reduce the success rate. Adhesion strength varies by case, and lead breakage risk depends on factors such as the lead type and extraction system used.

Thus, to explore methods for strengthening the rail, consisting of the lead and locking stylet, we measured the strength of each lead.

### Technique

Pacemaker leads have a thick IS-1 portion, which often is cut to reduce the powered sheath diameter during TLE, leading to the terminal's removal. However, the durability of the INGEVITY lead reportedly improves when its terminal is left intact or a snare is used during extraction.^12^ However, data on lead durability remain limited. To address this, we conducted verification tests on multiple leads under varying conditions.

Although individual variability was observed, all leads collectively elongated more with less force when the terminal pin was removed. Retaining the terminal likely reduces elongation by evenly distributing traction forces across the cathode and anode coils and insulation, dispersing stress. In contrast, when the terminal is removed, forces concentrate on central components, such as the cathode coil, weakening the lead. This leads to faster deformation in weaker areas, creating a cycle of stress and elongation. However, retaining the terminal is generally associated with an increase in lead diameter.

Grasping both the lead and locking stylet with a snare and buckling them into the sheath, as done in this study, is a critical technique for lead extraction.^12^ Leads grasped with a snare demonstrated increased durability. X-ray imaging revealed that coil structures remained more intact with a snare than without. The locking stylet enhances tensile strength and stabilizes the lead, and using a snare may amplify this by preventing stylet slippage.^13^

In addition, snare grasping proved beneficial across multiple lead types. It not only reinforced the lead but also facilitated disengagement from the vessel wall, improving both safety and procedural success.^14^, ^15^, ^16^ Although this technique increases complexity and procedure time, it should be considered for challenging cases.

Finally, we determined the One-Tie to provide a stronger binding force between the lead and locking stylet than suture material. Although suture material allows parallel traction to distribute force, this was not feasible in our setup. Thus, we used the One-Tie to better align with the conditions of our model.

### Lead-specific characteristics

Leads are composed of multiple components, such as coils and insulation, which result in a complex force curve during traction.^17^ The less pliable sections of the lead tend to rupture first, often accompanied by a snapping or popping sound, which is marked by a sudden decrease in the force required to pull the lead. The remaining elements then stretch, and phenomena such as inner coil unwinding can create an undulating pattern in the force curve. The average extractor-measured traction “pull” force during TLE has been reported to vary between 1.36 kgf (2.99 lbf) and 3.63 kgf (8.00 lbf).^12^ Based on our findings, we propose the following lead-specific considerations for when extracting pacemaker leads (Table 1).

**Table 1:** Maximal force for different extraction strategies and recommendations

Lead type	Maximum force (kgf)			Recommendation
	T-Rem	T-Ret	Snare	
INGEVITY	1.2	0.7	8.9	Use a snare
INGEVITY+	2.7	3.4	13.2	Retain the terminal Use a snare if higher force anticipated
Tendril STS	2.7	5.7	7.8	Retain the terminal Use a snare if higher force anticipated
Solia S	3.4	4.9		Remove terminal Retain terminal if higher force anticipated
CapSure Fix	5.9	6.2		Remove the terminal

T-Rem = terminal removed; T-Ret = terminal retained.

#### Ingevity/ingevity+

The INGEVITY lead exhibited weak durability irrespective of whether the terminal was removed or retained, although its durability increased when it was secured with a snare. The INGEVITY+ lead demonstrated enhanced durability when the terminal was retained. This change may be due to the structural change in the INGEVITY+ lead, which uses a triple-wound rather than a single-wound inner coil.^18^^,^^19^ Our findings suggest that a snare should be used in most TLE cases involving an INGEVITY lead. For the INGEVITY+ lead, the terminal should be retained and a snare considered if higher traction forces are anticipated.

#### Tendril STS

The Tendril STS lead exhibited weak durability when the terminal was removed. However, it demonstrated durability comparable to the Solia S and CapSure Fix leads when the terminal was retained. The use of a snare increased its strength even more, allowing it to endure forces of up to 7.8 kgf (17.2 lbf). Our findings suggest that the lead terminal ideally should be retained during TLE of a Tendril STS lead, and if a high traction force is anticipated because of clinical characteristics, a snare should be considered as an initial approach or adopted early during lead extraction.

#### CapSure Fix/Solia S

Both the Solia S and CapSure Fix had similar early behavior, with more force required before elongation. Both leads demonstrated greater strength than the other leads. The Solia S withstood 3.4 kgf (7.5 lbf) when the terminal was removed and 4.9 kgf (10.8 lbf) when the terminal was retained, whereas the CapSure Fix withstood 5.9 kgf (13.0 lbf) when the terminal was removed and 6.2 kgf (13.67 lbf) when the terminal was retained. The Solia S lead had the least amount of sliding of the locking stylet of any of the tested leads regardless of whether the terminal removed or retained (Figures 7C and 7D). Terminal removal to reduce the sheath diameter might be the best initial approach for TLE of these leads.

### Study limitations

In clinical practice, TLE complexity often increases with passive and older leads, but we focused exclusively on new active fixation leads, leaving the variability associated with aging leads and the challenges posed by passive leads unaddressed. Additionally, this was an *in vitro* study, and whether lead behavior would be similar *in vivo* is unclear because potential changes in durability due to aging were not examined. Furthermore, we used a straightforward lead traction test without incorporating simulated heart models. In the terminal-removed group, manual lead cutting introduced potential variability. Because of the limited number of available leads, we assessed only 1 lead per condition; thus, potential variability among leads within the same family was not evaluated. For the same reason, we were unable to conduct snaring tests on the Solia and CapSure leads. Moreover, this study investigated the use of only a single type of lead locking stylet, the Liberator. Exploring additional stylet types in future studies may provide further insights. Fluoroscopy was used pre- and post-traction testing, with video recordings during the process, but detailed examination of lead components during testing was not performed.

## Conclusion

Lead traction experiments were conducted to help predict the durability of leads during TLE and demonstrated different characteristics when subjected to standardized loading conditions. Behavior varied among leads from different manufacturers that also changed based on specific extraction techniques. Generally, terminal retention increased lead durability, whereas snare grasping further enhanced the durability. In addition to clinical characteristics, our study shows the importance of considering potential differences in response to tensile force among lead types when planning and performing TLE.
